# Intelligent Pectin and Alginate-Based Biopolymeric Film Enriched with Purple Basil Anthocyanins for pH-Sensitive Detection of Chicken Meat Freshness

**DOI:** 10.1021/acsomega.5c01438

**Published:** 2025-04-29

**Authors:** Veselina Adımcılar, Emrah Torlak, F. Bedia Erim

**Affiliations:** 1 Department of Chemistry, 52971 Istanbul Technical University, Maslak, Istanbul 34469, Turkey; 2 Department of Molecular Biology and Genetics, 226846 Necmettin Erbakan University, Meram, Konya 42090, Turkey

## Abstract

Materials derived from renewable and natural resources, such as biopolymers, are increasingly favored over synthetic polymers in food packaging applications. Biopolymers integrated with bioactive compounds can eliminate the need for preservatives, providing nontoxic and safer alternatives. Furthermore, these biopolymers have the potential to facilitate real-time monitoring of food freshness through the incorporation of pH-sensitive natural extracts. In this study, a composite biopolymeric film was fabricated using alginate and pectin, with the film’s properties further enhanced by the addition of purple basil extract. The film’s indicator functionality was evaluated by monitoring the spoilage of skinless chicken breasts under refrigerated conditions. A visual color change was observed in the film, driven by the pH-sensitive properties of the purple basil extract, which corresponded with the progression of food spoilage. The resulting material presents a safe, edible, and cost-effective alternative to conventional packaging, paving the way for more sustainable and secure food packaging solutions.

## Graphical abstract

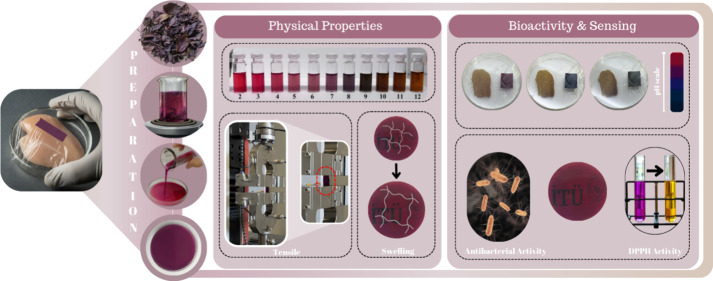

## Introduction

Reducing the decay rate of food and extending its shelf life are key priorities in the development of food packaging materials. Modern consumers increasingly demand packaging that not only preserves food but also serves as an indicator of its quality and freshness and playing a crucial role in their purchasing decisions.^1,2^ Furthermore, concerns about negative health effects, pollution, and the use of limited resources associated with conventional plastic packaging have been increasingly raised.^3,4^ Growing concerns regarding food safety and the environmental impact of using synthetic packaging have led to a shift toward sustainable, eco-friendly materials and the incorporation of natural resources for food preservation. These materials are categorized as active and intelligent packaging, which can signal food spoilage through visual color changes while simultaneously delaying deterioration and maintaining the nutritional value of the food.^5–8^ Since pH variations are a hallmark of food spoilage, anthocyanins, natural pigments, can act as effective indicators, altering their structure and color in response to changes in pH.^9–14^

The genus *Ocimum* encompasses 30 species with diverse flower colors, morphological traits, and chemical compositions. Among these, basil (Ocimum basilicum L.) from the Lamiaceae family is particularly noteworthy.^15^ Basil, valued both as a medicinal herb and as a culinary ingredient, is rich in essential oils, polyphenols, flavonoids, phenolic acids, and anthocyanins. Purple basil (O. basilicum purpurascens) is especially prominent for its high anthocyanin content, which causes visible color changes across various pH levels. In addition to its pH sensitivity, basil exhibits potent antioxidant, antimicrobial, antiviral, and antifungal properties.^16,17^ Such beneficial properties are highly desirable for developing of various types of novel active and intelligent food packaging.^18–25^

In response to environmental and health concerns, biopolymers have emerged as a promising alternative to conventional materials and also offer biodegradable and environmentally friendly solutions for food packaging. Biopolymers, derived from both synthetic and natural sources, offer minimal environmental impact and are being investigated for applications such as food packaging.^26^ Biopolymers such as chitosan, starch, alginate, and pectin are renewable, edible, biodegradable, and easily processable, making them viable alternatives to conventional petroleum-based plastics.

Pectin, a polysaccharide biopolymer extracted from plant cell walls, is primarily derived from the pulp and peels of citrus fruits (lemons, oranges, limes) and apples, which are the main sources for commercial pectin production.^27^ Known for its gelling and stabilizing properties, pectin is widely used in the food industry.^28^ Its chemical structure consists mainly of (1–4)-linked α-d-galacturonic acid units, with some esterified carboxyl groups.^28,29^

Alginate, a well-known linear anionic biopolymer extracted from brown seaweed, consists of alternating blocks of (1–4)-linked β-d-mannuronate and l-guluronate units.^28^ Alginate is valued for its film-forming ability, low toxicity, biodegradability, biocompatibility, and water solubility, making it a versatile material for food-related applications, including as a stabilizer, thickener, and binding agent.^30,31^

This study aims to develop an active and intelligent biopolymeric packaging material incorporating a natural source of anthocyanins. While composite films made from pectin and alginate have been investigated in a limited number of studies, such as those by Da Silva et al.,^32^ who studied the effect of plasticizer concentration, and by Galus and Lenart^33^ and Makaremi et al.,^28^ who reported on the physical properties of such films, there is a lack of research on their application with anthocyanin-rich extracts. Lei et al.^34^ explored films composed of pectin, sodium alginate, and cellulose nanocrystals, while Yong and Liu^35^ reviewed the physical and functional properties of anthocyanin-incorporated films.

Some studies have investigated the potential of basil extract in biopolymer-based films, including Medina-Jaramillo et al.,^36^ who reported on cassava starch films with basil extract, and Singh et al.,^37^ who explored the antimicrobial and antioxidant properties of poly­(vinyl alcohol) and cellulose films with basil extract. Furthermore, Ebrahimi and co-workers^38^ developed a gelatin-based pH-sensitive indicator film using purple basil leaf extract, which is rich in anthocyanins. Shakouri and co-workers^39^ reported on the preparation of the anthocyanin-loaded films with basil seed gum as a colorimetric indicator and incorporated with saffron petal extract for antioxidant activity enhancement. Barkhordri et al.^40^ developed a biobased gelatin film with nano-TiO_2_ and purple basil extract for spoilage detection. Moreover, Granados-Balbuena and co-workers summarized the whole patented applications related to anthocyanins in food.^41^

To the best of our knowledge, this study exploits the synergistic effects of anthocyanin-rich extract, enabling real-time spoilage detection with alginate pectin composite film containing purple bacillus extract for the first time while demonstrating this film’s improved antioxidant, antimicrobial, swelling, and solubility properties. These findings bring the simply prepared biopolymer-based smart packaging closer to real food applications. Our study highlights its potential as a multifunctional active food packaging material and an effective indicator for monitoring food freshness through a comprehensive evaluation of the film’s physical, antimicrobial, and antioxidant properties.

## Experimental Section

### Chemicals

Pectin citrus (yellow to pale-brown powder, galacturonic acid units ≥74.0%, and methoxy groups ≥6.7%) was purchased from Alfa-Aesar (Haverhill, Massachusetts, USA). Alginic acid sodium salt from brown algae (white to brown powder, medium viscosity ≥2000 cP, 2% (25 °C)), Folin–Ciocalteu reagent, gallic acid, 2,2-diphenyl-1-picrylhydrazyl (DPPH), 2,4,6-tripyridyl-*s*-triazine (TPTZ), and ferric chloride hexahydrate (FeCl_3_·6H_2_O) was purchased from Sigma-Aldrich (St. Louis, Missouri, USA). Glycerol (≥99.5%), calcium chloride dihydrate, sodium acetate trihydrate, sodium carbonate, sodium hydroxide, potassium chloride, methanol, hydrochloric acid, acetic acid, and ferrous sulfate heptahydrate (FeSO_4_·7H_2_O) were purchased from Merck (Darmstadt, Germany). A commercially available package of dried leaves of purple basil (Ahmet Arifoğlu) was purchased from a local herb market. Lyophilized cultures of Escherichia coli (ATCC 25922) and Staphylococcus aureus (ATCC 6538) were supplied from Liofilchem (Roseto degli Abruzzi, Italy). All reagents were analytical grade and were used as received. Distilled water was obtained by the ELGA PURELAB Option-7-15 model system with 18.2 Ω resistivity.

### 2.2 Preparation of Purple Basil Extracts

Dried leaves of purple basil were finely ground with a laboratory mill. Ten grams of the leaves was extracted with 100 mL of freshly boiled deionized water. The solution was incubated for 5 min in a water bath and then filtered through Whatman No. 41 filter paper. The extract was prepared daily and kept in the laboratory refrigerator wrapped with aluminum foil until use for analysis and film preparation.

### 2.3 Preparation of Biopolymeric Films

Biopolymeric composite films were prepared by using two different biopolymers, pectin and alginate. For all film-forming solutions, citrus pectin at a final concentration of 1% (w/v) was dissolved in 1:1 (v/v) diluted purple basil extract with water for the intelligent film. Each film-forming solution was allowed constant stirring at 300 rpm on a magnetic stirrer of IKA (Staufen, Germany) for 2 h at room temperature. Then, the amount of sodium alginate corresponding to 0.5% (w/v) was weighed and added to the mixture, and the mixture was stirred for 2 h more. 350 μL of glycerol was added to 30 mL of a film-forming solution and allowed to stir for an hour. The film-forming solutions, including purple basil extract, were wrapped with aluminum foil during stirring. The control film sample without an extract was prepared using all components and procedures described above. Then, all of the film solutions were poured into 9 cm plastic Petri dishes and allowed to dry homogeneously in an oven (Nüve EV 018, Ankara, Turkey) for 48 h at 40 °C.

The dried films, after casting, were not physically stable when they were placed in water. The films disintegrated and solubilized, proving that they are unsuitable for food-related applications. For this purpose, the films were subjected to ionic cross-linking to produce films with higher water resistance. The dried films were immersed in 30 mL of 3% (w/v) CaCl_2_ solution for 15 min. Then, the films were washed with distilled water to remove unreacted CaCl_2_, left to dry at room temperature for 12 h, and used for further analysis.

### 2.4 Measurement of Film Thickness, Opacity, and Light Transparency

A hand-held digital caliper with 0–150 mm sensitivity was used for the measurement of the thickness of the prepared film samples as the average value of five different measurements from random points of each film. The average film thickness for each film was used in further calculations.

The films’ light transmission was assessed using the Shimadzu UV-1800 model UV–Vis spectrophotometer from Kyoto, Japan. The films were cut into 2 × 2.5 cm^2^ pieces, and the transmittance spectrum of each film was recorded within the wavelength range 200–800 nm. An empty cell containing air was used as a reference. Opacity values were determined at 600 nm using the average film thickness (*d*) and the absorbance (*A*) values, calculated using the equation below.

$$\mathrm{Opacity}\;600=\frac{\mathrm{Abs}\;600}{d}$$ 1

### 2.5 Fourier Transform Infrared Spectroscopic Analysis

Both films’ Fourier transform infrared (FTIR) spectroscopic analysis was conducted using an Agilent Cary 630 FTIR Spectrometer from California, USA. The transmittance spectra of the films were measured in the wavenumber range of 4000 to 400 cm^–1^, with a resolution of 4 cm^–1^ and 64 scans.

### 2.6 Differential Scanning Calorimetry Analysis

The thermal properties of the films prepared with and without purple basil extract were comparatively evaluated by using differential scanning calorimetry (DSC). The analysis was performed on a PerkinElmer Diamond DSC instrument (Waltham, Massachusetts, USA). Film samples, weighing approximately 3–4 mg, were heated over the temperature range 30–330 °C at a heating rate of 10 °C/min. A nitrogen (N_2_) atmosphere was maintained throughout the analysis with a flow rate of 20 mL/min to ensure an inert environment, and thermograms were recorded.

### 2.7 Evaluation of the Mechanical Properties of the Films

The mechanical properties of both control and purple basil enriched films were investigated by using a Zwick-Roell testing device (Ulm, Germany) attached to a 500 N force load cell. All measurements were carried out at a speed of 10 mm/min. The film samples were cut into rectangular dimensions, measured, and placed between jaws at a distance of 15 mm. All measurements were performed under room-temperature and humidity conditions and performed in three replicates. The tensile strength (TS), Young’s modulus (YM), and elongation at break (EB %) values were calculated from the stress–strain curves.

### 2.8 Determination of Surface pH of the Films

The surface pH of the biopolymeric films was determined at a room temperature of 25 °C. The film surfaces were slightly wetted with distilled water, and the MColorpHast Merck (Darmstadt, Germany) pH indicator strips were used.^42^ The strips were placed on the wet surface of each film until they were moist enough, and the pH value was determined and recorded by the corresponding color change on the strip.

### 2.9 Determination of the Moisture Content of the Films

The films’ moisture contents (MC) were determined using the Shimadzu MOC63U UniBloc Moisture Analyzer (Kyoto, Japan). Each sample was cut into 2 × 2 cm^2^ pieces, placed on the balance of the moisture analyzer, and heated up to 105 °C with a constant rate of 1 °C increment of 30 s. When the difference between masses reached 0.03%, moisture content was recorded.^42^ Measurements were triplicated for each sample.

### 2.10 Determination of Water Vapor Transmission Rate and Water Vapor Permeability

The water vapor permeabilities (WVP) and the water vapor transmission rate (WVTR) of the control and the purple basil incorporated films were evaluated gravimetrically according to the ASTM E96-95 “Water Method” with slight modifications presented as in our previous work.^42^ Both types of films were cut into circular shapes and then tightly mounted on glass bottles containing distilled water 2 cm below the top. Each bottle was delicately weighed before and then placed in a desiccator at 25 °C preconditioned to 75% humidity using a saturated NaCl solution. The bottles were weighed with an electrical balance for definite time intervals (Mettler Toledo, tare range: 0–100 g; sensitivity: 0.00001, Ohio, USA). The curve between the loss of weight and the time was drawn. Linear regression was used to obtain the slope. The WVTR was calculated according to eq 2.

$$\mathrm{WVTR}=\frac{m}{A}$$ 2

where *m* represents the slope and *A* is the area of the films subjected to the water vapor transmission. The WVP of each film, proportional to their thickness (*d*) in millimeters, was reported according to eq 3, where Δ*P* is the water vapor partial pressure difference on two sides. The results are expressed as the mean value of three samples taken from each film.

$$\mathrm{WVP}=\left(\frac{\mathrm{WVTR}}{\Delta P}\right)\times d$$ 3

### 2.11 Determination of the Swelling Properties of the Films

The swelling properties of each film were determined in distilled water at 25 °C. For this purpose, the films were taken, cut into 2 × 2 cm^2^ small pieces, and thoroughly dried in an oven (Nüve EV 018, Ankara, Türkiye) at 70 °C for 24 h. Then, the initial weighing of the films was recorded, and films were rapidly immersed in 50 mL of distilled water-containing beakers at 25 °C placed in a Nüve ST-402 shaking water bath (Ankara, Turkey). The beakers were shaken at a constant rate of 50 rpm. At predetermined time intervals (1, 5, 15, 30, 45, and 60 min to 2, 3, and 24 h), films were taken from the water, and excess surface water on the films was dried gently with filter paper and carefully weighed. All measurements were conducted with three samples for each film. The swelling ratio percentages (SR %) were calculated according to eq 4.

$$\mathrm{SR}\%=\frac{M_{\mathrm{final}}-M_{\mathrm{initial}}}{M_{\mathrm{initial}}}\times 100$$ 4

*M*_Initial_ and *M*_Final_ are masses of films before and after swelling.

### 2.12 Determination of the Total Water Solubility of the Prepared Films

To assess the physical integrity of the films toward food with water content, the total water solubility (TWS) of each film was determined. The prepared films were taken and cut into 2 × 2 cm^2^ pieces and thoroughly dried in an oven at 70 °C for 24 h (Nüve EV 018, Ankara, Türkiye). Then, the initial weighing of the films was recorded, and films were immersed in 50 mL of distilled water-containing beakers at 25 °C placed in a Nüve ST-402 shaking water bath (Ankara, Turkey) and shaken at a constant rate of 50 rpm for 24 h. Then, the pieces were carefully taken and dried in the oven at 70 °C for another 24 h to allow completely dry. Then, the films were reweighed. The difference between the masses was calculated using eq 5, and the total water solubility % values were recorded.

$$\mathrm{TWS}\%=\left(\frac{M_{2}-M_{1}}{M_{1}}\right)\times 100$$ 5

*M*_1_ and *M*_2_ are the masses of the films. *M*_1_ is the dried film before immersion in water, and *M*_2_ is the dried film after immersion in water.

### 2.13 Determination of Total Phenolic Content (TPC), Antioxidant Activities, and Total Anthocyanin Amounts (TAA) and TAA Release of Purple Basil and Film Extracts

Since purple basil extract in biopolymers was expected to create an active food coating material thanks to the extract’s bioactive compounds, the extracts’ total phenolic amounts, antioxidant activities, and total anthocyanin amounts were checked first. The prepared film’s expected intelligent film feature is that it responds to food spoilage by changing color, which will depend on the anthocyanin amount of the extracted content. The release of TAA with time into the water environment was monitored to determine the decrease in the total anthocyanin amount in the extract added to the biopolymeric films. In this way, the presence of anthocyanins that remained unreleased in the film and served as indicators for food spoilage was demonstrated.

The TPC contents were evaluated based on the well-known Folin–Ciocalteu method that Singleton and Rossi^43^ developed with minor modifications. 300 μL of diluted purple basil extract was mixed with 1.5 mL of 1:10 diluted Folin–Ciocalteu’s reagent and 1.2 mL of %7.5 (w/v) aqueous sodium carbonate solutions. The mixture was allowed to stand for 15 min at room temperature, and the absorbance was measured at 760 nm against blank distilled water. All experiments were duplicated. Total phenolic content was calculated from the five-point calibration curve constructed with standard gallic acid (GA) solutions. The results were expressed as milligrams of gallic acid equivalents (GAE)/g of dry weight (DW) of purple basil.

Antioxidant active food packaging protects against oxidation by scavenging harmful free radicals and preventing food from spoiling. Therefore, to prove that the diffusion of the prepared packaging into foods, the total amount of phenolics and antioxidants released from the doped biopolymeric film into the water was determined. 10 mg of film samples was extracted in 5 mL of deionized water, and the mixture was vortexed for a minute and then ultrasonicated for 20 min. 300 μL of purple basil film extract was mixed with Folin–Ciocalteu’s reagent and aqueous sodium carbonate solutions similar to that performed for purple basil extract, and TPC of the film was calculated as mg of gallic acid equivalents (GAE)/g DW of the film sample. Thus, the feature of the prepared films as active food coating material was demonstrated

The FRAP assay followed the established protocol by Benzie and Strain.^44^ 10 mg of the film samples was extracted in 15 mL of distilled water. The mixture was vortexed for a minute and then ultrasonicated for 20 min at room temperature. A 100 μL volume of the film extract or diluted purple basil extract was mixed with 100 μL of distilled water and 1800 μL of the FRAP solution, and after incubation for 15 min in a water bath at 37 °C, the absorbance values were recorded at 593 nm against distilled water. The results were expressed as mg Fe­(II)/g DW of film sample or mg Fe­(II)/g DW purple basil according to the calibration curve constructed using FeSO_4_ as a standard. All experiments were performed in duplicates.

For the evaluation of the radical scavenging activities of the purple basil extract, film extracts at different concentrations were determined using the DPPH method. The 50 μL from the extract solutions was mixed with 1950 μL of the methanolic DPPH solution, vortexed, and kept in the dark for 30 min, and the absorbance values of the solutions were measured at 517 nm against methanol. The antioxidant activity reported as inhibition % was calculated according to eq 6. All experiments were duplicated.

$$\mathrm{Inhibition}\%=\frac{\mathrm{Abs}\mathrm{control}-\mathrm{Abs}\mathrm{sample}}{\mathrm{Abs}\mathrm{control}}$$ 6

“Abs control” is the mixture prepared by using distilled water as extract, and “Abs sample” is the absorbance of the solutions prepared with extracts and DPPH.

Purple basil extracts’ total anthocyanin amount (TAA) was determined as milligrams per liter, using the pH differential method. Anthocyanin pigments change their structures with pH; thus, the absorbance spectra change. The color differences were recorded by using UV–Vis spectroscopy. Absorption values of the extracts were determined for two different pH values, 1.0 and 4.5, at 516 and 700 nm, respectively. Potassium chloride buffer at pH 1.0 and sodium acetate buffer at pH 4.5 were used. The results were expressed as cyanidin-3-glucoside (Cy-3-glc) equivalents by using eqs 7 and 8.

Abs=(Abs516−Abs700)pH1.0−(Abs516−Abs700)pH4.5 7

$$\mathrm{TAA}=\frac{\mathrm{Abs}\times \mathrm{MW}\times \mathrm{DF}\times 1000}{\varepsilon \times l}$$ 8

where MW is the molecular weight, DF is the total dilution factor, *l* is the path length, and MW is molecular weight and ε the molar absorptivity of cyanidin-3-glucoside, which are MW = 449.2 g/mol and ε = 26,900 (L/mol·cm), respectively.

To determine the released amount of anthocyanin with time, 30 mg of film samples was placed in a 50 mL distilled water-containing beaker wrapped with an aluminum foil and shaken at a constant rate of 150 rpm with an IKA KS 260 (Staufen, Germany) orbital shaker at room temperature. The pH values of 2 mL solutions from the release medium were adjusted to 1.0 and 4.5, respectively. Distilled water was added to as much as the amount withdrawn from the solutions. TAA was calculated from the measured absorbance values of the solutions with the help of eqs 7 and 8.

The release rate of anthocyanin from the film was calculated using eq 9. The experiment was duplicated.

$$\mathrm{TAA}\;\mathrm{release}\%=\frac{M_{t}}{M_{i}}\times 100$$ 9

where *M*_t_ is the amount of calculated TAA released at predetermined times (5 min, 15 min, 30 min, 1 h, 2 h, 4 h, and 6 h), and *M*_i_ is the TAA amount of the film calculated from initially added purple basil extract.

### 2.14 Evaluation of the Antibacterial Activity of the Prepared Film

Antibacterial activities of films were determined by quantifying the survival of bacteria held in intimate contact with film samples according to the ISO 22196 standard.^45^ Two microorganism species representing Gram negatives and positives were used in the antibacterial assay of the films. Stock cultures of microorganisms were stored in Brain Heart Infusion Broth (Liofilchem) supplemented with 20% glycerol at −18 °C. Working cultures were grown on Nutrient Agar (Liofilchem) slants. The overnight slant cultures were transferred in 1/500 Nutrient Broth (Liofilchem), and the cell density of suspensions was adjusted to the 0.5 McFarland turbidity standard, which represents approximately 1.5 × 108 colony forming units (CFU)/mL. These suspensions were diluted in 1/500 Nutrient Broth to obtain a working concentration of about 1.0 × 106 CFU/mL.

Test film samples of 50 × 50 mm square dimensions, sterilized under UV–light, were placed in separate sterile Petri dishes, and 200 μL of test inoculum of microorganisms was pipetted onto the film samples. Then, the inoculated film samples were covered with sterilized plain polypropylene (PP) film pieces of 40 mm × 40 mm to spread the inoculum on the test films. Sterile plain PP film pieces of the same size as the film samples were used as controls. Petri dishes contained the inoculated test and control films for 6 h at 35 °C under a relative humidity of above 90%.

The numbers of viable microorganisms on the test and control films were determined by a plate count technique immediately after inoculation and after 6 h of contact time. The microorganisms on the films were recovered in D/E Neutralizing Broth (Acumedia, Lansing, USA). Then, D/E Neutralizing Broth and its decimal dilutions prepared with phosphate-buffered physiological saline were plated on Plate Count Agar (Liofilchem), and the inoculated plates were incubated at 35 °C for 48 h. The resultant colonies were counted, and the number of viable microorganisms was calculated as log CFU/sample.

### 2.15 Determination of Color Changes of the Purple Basil Extract and Film Samples with pH

Color changes of the purple basil extracts were observed in the pH range of 2.0 to 12.0 by adjusting the desired pH level by adding HCl or NaOH solutions, and images were recorded. The UV–Visible spectra of the extracts were measured using a Shimadzu UV-1800 model UV–Vis spectrophotometer (Kyoto, Japan) against ultrapure water as the blank solution.

The prepared films were placed in several buffer solutions with different pH values (2.0, 4.0, 6.0, 8.0, 10.0, and 12.0), where distinct and strong color changes were observed, and images were recorded.

### 2.16 Application of the Films to a Real Food Sample and Determination of Changes in Color Properties

The developed film was tested for its applicability as an indicator of food freshness. As a protein-rich food, fresh chicken breast meat was used for testing purposes. A 4 × 4 cm^2^ square piece of film containing purple basil extract was placed inside the package without touching the chicken breast, and the mouth of the Petri dish was closed. The visual observations for chicken breast meat and the film sample indicating the freshness of the product were evaluated at 4 °C storage condition. After the change in the color of the film concerning the deterioration of the food, color properties were measured by using white Lenata cards from three different positions of each film piece. Average values were calculated, and the total color change of the films was also calculated by using the eq 10 given below.

$$\Delta E=\sqrt{{\left(L^{*}2-L^{*}1\right)}^{2}+{\left(a^{*}2-a^{*}1\right)}^{2}+{\left(b^{*}2-b^{*}1\right)}^{2}}$$ 10

where L*_1_, a*_1_, and b*_1_ are reference color parameters and L*_2_, a*_2_, and b*_2_ for another color gives the Δ*E* color change.

## Results and Discussion

### 3.1 Optimization of the Film Formulations

The film-forming solutions, prepared as described in Section 2.3, with and without purple basil extract, were poured into plastic Petri dishes in 30 mL quantities. The intense purple color of the basil extract restricted the amount used in the formulation of the prepared film. An increase in the amount of extract in the film-forming solution resulted in an intense dark color in the obtained film. Thus, the 1:1 (v/v) diluted purple basil extract was selected as the composition for film-forming solution. The amount of pectin between 0.5 and 2% (w/v) was tested, and 1% was selected as the optimum concentration. Below this level, the films were not easy to handle. The further increase in the concentration of pectin increased the film thickness. The durability of the films was optimized with the addition of sodium alginate to the film-forming solution. A sodium alginate of 0.5% (w/v) concentration was selected in the film-forming solution. Further increase in the alginate concentration did not result in a homogeneous film formulation. Glycerol was used as a plasticizer and added to the film-forming solution at a 1:1 polymer weight ratio. As explained in the experimental part, the film-forming solution was stirred after each addition at room temperature on a magnetic stirrer at a stirring rate of 300 rpm. Then, the films were poured into 9 cm plastic Petri dishes.

After drying of the films, the physical endurance in water was investigated. Films were placed in a beaker containing distilled water, and dissolution of the films was observed, proving that they are not suitable for use in food-included applications without cross-linking. Pectin and alginate natural polymers, which have polyuronate structures, form hydrogels with divalent metal ions.^46^ The most used cation for this purpose is the Ca^2+^ ions. For this purpose, the films were subjected to ionic cross-linking using a 3% (w/v) CaCl_2_ solution. Dried films were immersed in 30 mL of CaCl_2_ solution for 15 min and then washed with deionized water to remove the excess cross-linker. Then, the films were air-dried at room temperature.

### 3.2 Film Thickness, Opacity, and Light Transparency

Adding purple basil extract to the pectin-alginate film formulation resulted in a slight decrease in the film thickness; the values are listed in Table 1. The amount and composition of anthocyanins affect the film thickness differently. The same trend was observed in films with anthocyanin-added cellulose acetate, as reported by Freitas et al.^47^ and anthocyanin-added corn starch films by Prietto et al.^48^ The opacity value of the film enriched with plant extract was increased significantly because of the natural purple color of the extract due to a group of pigments known as anthocyanins. It may be preferable for the packaging material to be opaque, especially to protect easily degradable foods.^49^ The opacity values are listed in Table 1. The transmittance spectra in the wavelength range 200–800 nm were measured to evaluate the UV–Vis light transmittance of the films. Both films showed a UV-light-blocking ability. The transmittance spectra of the films are given in Figure 1A. The digital photographs of both the control film and basil film are shown in Figure 1B.

**Table 1:** Physical and Optical Properties of the Prepared Biopolymeric Film Samples

physical properties	control film	purple basil film
thickness (mm)^b^	0.124 ± 0.024	0.074 ± 0.011
opacity (mm^–1^)^a^	0.77 ± 0.01	11.31 ± 0.04
moisture content %^c^	22.44 ± 1.18	18.07 ± 3.03
total water solubility %^a^	21.08 ± 2.07	32.38 ± 0.82
swelling %^a^	441 ± 26	777 ± 25
WVTR (g/day·m^2^)^a^	12.92 ± 0.02	20.79 ± 0.01
WVP (g·mm/day·m^2^·mmHg)^a^	0.067 ± 0.001	0.065 ± 0.001
tensile strength (MPa)^c^	103.38 ± 10.01	99.11 ± 10.87
Young modulus (MPa)^c^	39.91 ± 3.16	30.94 ± 4.08
elongation at break %^c^	3.63 ± 0.20	3.69 ± 0.28

a ± Standard deviations (SD), *n* = 3.

b ± Standard deviations (SD), *n* = 5.

c ± Standard deviations (SD), *n* = 2.

**Figure 1:**
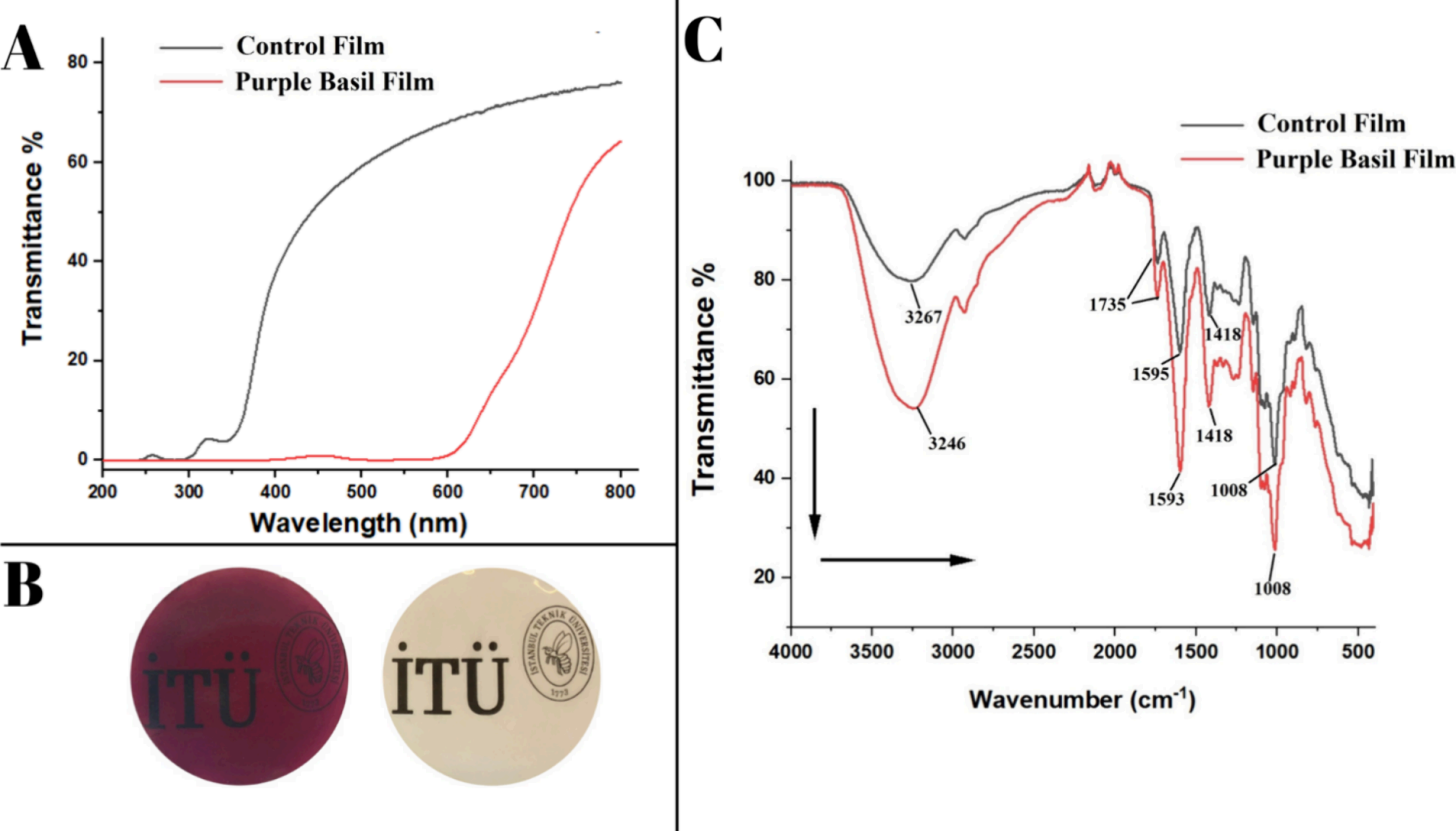
(A) UV–Vis transmittance spectra of the purple basil and the control film prepared without extract. (B) Digital photographs of the film samples: film with purple basil extract (left) and control film prepared without extract (right). (C) FTIR spectra of the purple basil film and the film prepared without extract.

### 3.3 Fourier Transform Infrared Spectroscopic Analysis

The FTIR spectra of the control film and the film prepared with basil extract after cross-linking are represented in Figure 1C. The O–H stretching vibration of the sole pectin-alginate film was obtained at the frequency of 3267 cm^–1^, asymmetric −COO^–^ stretching was obtained at 1595 cm^–1^, and symmetric −COO^–^ stretching was observed at 1418 cm^–1^. Also, the −C=O vibrations of the −COOCH_3_ group were observed at 1735 cm^–1^.^28^ The film obtained with the purple basil extract showed a similar spectrum, while the peak intensities were increased, and slight shifts were obtained for the O–H stretching and asymmetric −COO– stretching to shorter wavenumbers, 3246 and 1593 cm^–1^, respectively. The shifts and peak broadening indicate the interaction of the extract and polymer blend via hydrogen bonding. The FTIR spectra of the control film and the film with the extract also supported that the interaction between the extracted matrix and biopolymers is noncovalent due to the absence of major shifts in peaks and formation of new ones.^42,50^

### 3.4 Differential Scanning Calorimetry Analysis

The thermal properties of films with and without the extract (control film) were investigated. DSC curves of the films are shown in Figure 2. Both films showed endothermic peaks around 150–160 °C and an exothermic transition in the 200–280 °C range. The endothermic peaks between 80 and 160 °C are usually reported as moisture loss and polymer relaxation depending on the polymer matrix and the degree of cross-linking.^28,51^ The control film showed an endothermic peak around 100–150 °C suggesting moisture loss, while a slight shift to higher temperatures around 120–170 °C was obtained for the film prepared with the extract. This can be attributed to the interaction of the ingredients of the extract and the polymer chains, altering the polymer composite structure, which may reduce the number of free water molecules present in the structure.

**Figure 2:**
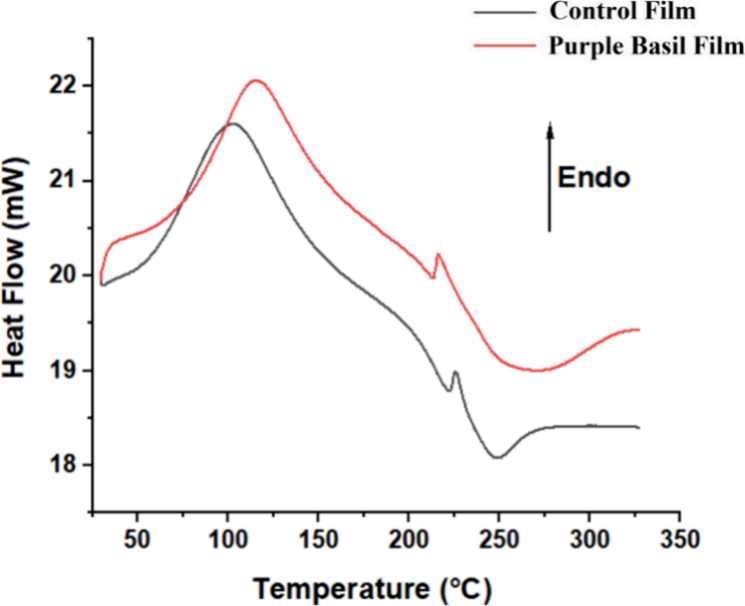
DSC thermogram of the purple basil film and the control film prepared without extract.

Exothermic peaks obtained indicate the possible polymer degradation and structural rearrangements due to Ca^2+^ cross-linking.^51^ Peaks obtained around 250–260 °C suggest partial alginate and pectin thermal decomposition. The film with the purple basil extract showed a lower onset degradation temperature, meaning that the extract may only slightly reduce thermal stability due to the changes in structure. At a temperature of 300 °C, stabilization of the curves suggested the beginning of the major degradation of polymers. The film with the extract showed a more stable end where the onset temperature of major decomposition obtained 320–350 °C, possibly due to antioxidant active ingredients present in the extract.^35^ The remaining strong final thermal stability proved that both films could withstand moderate processing temperatures. The DSC thermograms confirmed the moderate stability of the materials, and they are promising for use in food packaging.

### 3.5 Evaluation of the Mechanical Properties of the Films

The differences in the mechanical properties of the biopolymers can elucidate the various interactions among components and additives. To compare the mechanical properties, the TS, Young’s modulus (E), and elongation at break (EB) % values of both films were tested. TS indicates a material’s resistance to breaking under tension. At the same time, Young’s modulus is a specific type of elastic modulus that indicates the stiffness or elasticity of a material under stress. All mechanical test results are presented in Table 1. The tensile strengths of natural biopolymers vary according to their origin, concentrations, and cross-linking properties. The composite film prepared in this study gave a high TS value. The fact that alginate and pectin are compatible polymers and the strong cross-linking properties of each with calcium ions are the reasons for the high TS of the composite film.^30^ TS values in Table 1 are compatible with the TS values of alginate-pectin films reported by Bierhalz et al.^52^ The TS value of the purple basil film decreased compared to that of the control film. This situation has been observed in the literature in alginate-pectin films with bioactive substances added and interpreted as the additional active substances interacting with the hydrogen bonds of the polymer chains, reducing the interaction between the chains, and some chains cross-linked with Ca^2+^ ions cause breaks. On the other hand, adding the purple basil extract to the films did not significantly affect the elongation at break (EB). EB% only measures the ability of a material to stretch before breaking, does not represent overall strength or durability, and does not necessarily correlate with TS or YM for blend films.^53^ A similar situation was observed in alginate-pectin films with the addition of natamycin.^52^ Similar trends were reported in some other reports investigating the addition of tea polyphenols or grapefruit seed extract into biopolymer structures.^54,55^ However, the film incorporated with purple basil extract still has enough strength and high flexibility due to the decrease in the YM from 39.91 to 30.94 MPa. In this case, the addition of purple basil extract into the biopolymer structure resulted in the disruption of strong interactions between polymer chains; on the other hand, the extract successfully acted as a plasticizer by allowing them to move more freely and made the film flexible.^56^ In our study, the TS and YM values indicate that the material has good TS and flexibility for food-related applications.

### 3.6 Surface pH of the Films

The surface pH of the film prepared with basil extract was 3. Both pectin and alginate are acidic biopolymers. The pH of the basil extract was measured to be around 5. It is reported that the slightly acidic nature of the film increases its protective ability against bacterial growth.^57^

### 3.7 Moisture Content of the Films

The film prepared with the extract of purple basil showed a slight decrease in moisture content compared with that of the film prepared without the extract. The results are represented in Table 1. The reason for this is that the ability of the composite film to absorb water molecules from the air decreases due to the hydrogen bonds formed by the phenolic groups of the purple basil extract with the hydroxyl groups of the polymer composite, limiting the interaction between water molecules and the ionic groups of the biopolymers. Similar trends were reported for the films prepared with chitosan and some vinegar varieties^42^ and for the κ-carrageenan films with and mulberry polyphenolic extract.^58^

### 3.8 Water Vapor Transmission Rate and Water Vapor Permeability of the Films

Barrier properties of the films were also comparatively investigated, since the moisture transmission rate is considered a crucial parameter for the determination of the shelf life of a product. A slight increase was observed in the WVTR of the purple basil film, which can be attributed to the interaction of the extract matrix constituents with the biopolymers. The interaction slightly weakens the cross-linking between the polymer chains, meanwhile creating slight porosity in the structure, thus increasing WVTR.^42^ On the other hand, WVP slightly decreased since it is related to the thickness. The results are listed in Table 1.

### 3.9 Swelling Ability of the Films

Water intake of the prepared material was reported by determination of the swelling properties of both the control and the basil-included film, which was studied comparatively, and the results are shown in Figure 3 as swelling % over time. The total swelling % of both films after 24 h is also represented in Table 1. The swelling ability of the film prepared with basil extract increased 1.5 times compared to that prepared without extract. Basil is a plant rich in hydrophilic compounds such as anthocyanins, phenolic acids, and flavonoids where high polyphenol content of the extract is proved with the results showing the high TPC of the extract. Phenolic compounds have an affinity toward water molecules and are capable of forming hydrogen bonds, thus considered as the reason for the increased swelling.^59^ The incorporation of anthocyanins can also lead to structural modifications within the polymer structure, altering water-related physical properties, as reported.^60^ These interactions may result in a more flexible network, allowing for greater water absorption as a result of the interaction between the hydrophilic molecules and water.^61,62^ Similarly, a study reported that encapsulating basil seed oil in hydrogel beads made from sodium alginate and modified starch contributed to the beads’ water retention capacity due to the hydrophilic nature of basil components.^63^

**Figure 3:**
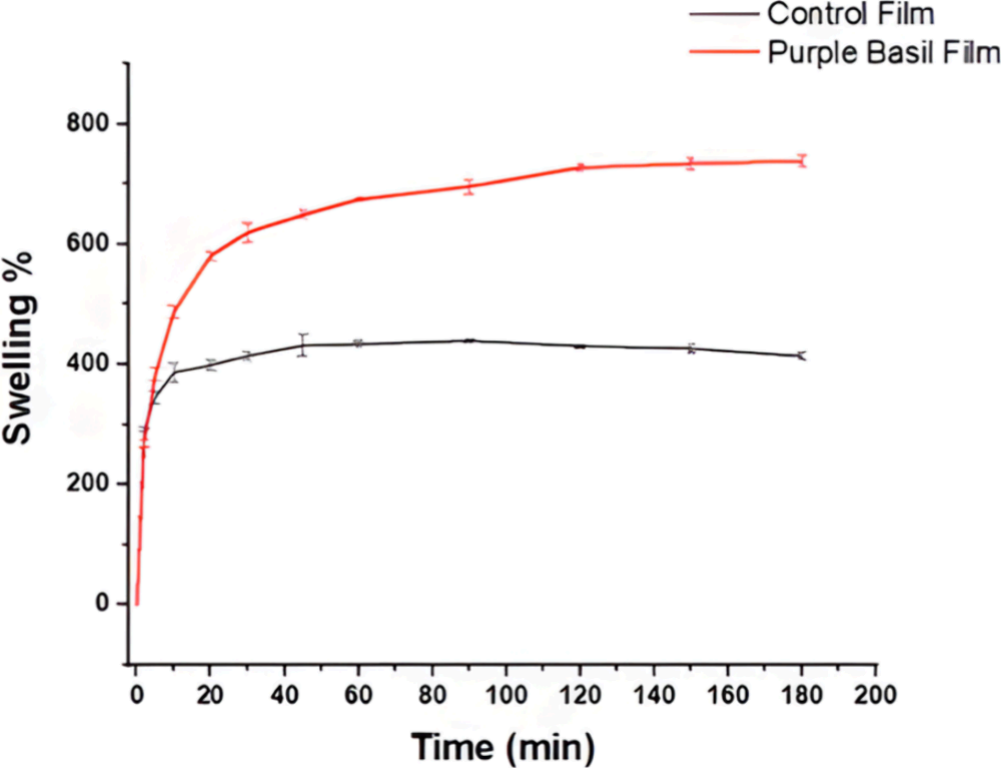
Swelling behavior of the purple basil film and the film prepared without extract.

### 3.10 Water Solubility of the Films

To assess the physical integrity of the films, the total water solubilities were determined, and the results are represented in Table 1. According to the findings, the films prepared with the purple basil extract showed a slightly higher water solubility than those without the extract. Since the extract is rich in natural plant base pigments, anthocyanins, and other organic acids that have an affinity toward water, the solubility increased. The results are consistent with the previous studies using anthocyanins as an additive in intelligent film preparation.^48,64,65^

### 3.11 TPC of the Purple Basil and Film Extracts

The TPC calculated for the extract was found to be 46.89 mg of GAE/g of DW purple basil. Hęś et al.^66^ reported TP contents of four different basil-type extracts in the range of 20.34–165.44 GAE/g DW. The plant’s TP composition may vary depending on its growing region and extraction conditions. The TP values obtained in this study are compatible with the TP values of the basil extracts obtained from the same area and reported by Gülhan et al.^67^ and Erez and Bayramoğlu.^68^ A high TPC value strongly indicates the rich polyphenolic content of the extract and its increased bioactive and protective properties.

The total phenolic content of the purple basil film extract was 24.11 ± 1.66 mg of GAE/g of DW film. In contrast to the control film, which showed no TPC, the basil film showed a significant TPC value.

### 3.12 Antioxidant Activities of the Purple Basil and Film Extracts

The antioxidant power of the purple basil extract and both film extracts was evaluated by using the DPPH method. The DPPH inhibition % value of the purple basil film extract and purple basil extract with different concentration levels are represented in Figure 4A and Figure 4B, respectively. The DPPH radical scavenging activity was found to be 89.85% at the concentration level of 6.60 μg of DW purple basil/mL, which strongly indicates that purple basil is one of the natural sources of antioxidants.

**Figure 4:**
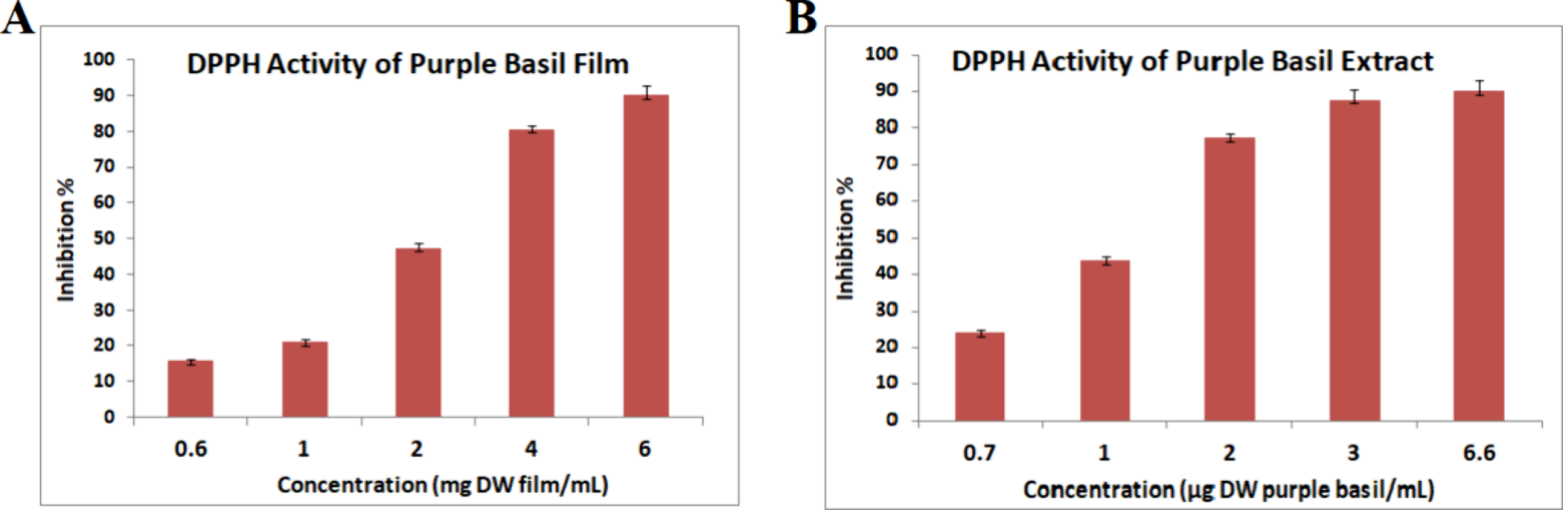
DPPH inhibition % of (A) purple basil film extract and (B) purple basil extract.

While the control film prepared without purple basil extract exhibited no antioxidant activity, the film prepared with the addition of purple basil extract demonstrated a high radical scavenging activity of 90.10% at a concentration level of 6 mg purple basil film/mL. Similar results indicating an increase in the antioxidant ability of the films following the addition of natural green tea extracts were reported by Siripatrawan and Harte,^69^ and Alizadeh-Sani et al.^70^ also reported that the addition of barberry anthocyanins to methylcellulose/chitosan-based films showed similar and improved DPPH scavenging activity.

The extract’s FRAP results indicate that dry purple basil has a robust ferric-reducing ability, with 257.22 ± 31.45 mg of Fe­(II)/g of DW purple basil. The film extract’s FRAP value was 238.04 ± 5.91 mg Fe­(II)/g DW film.

### 3.13 TAA of the Purple Basil Extract

As explained in the experimental part, the TAA of the basil extract was determined using the pH differential method. The total monomeric anthocyanin content of the purple basil extract was calculated as 4.87 ± 0.49 mg Cy-3-glc/g DW. The results were in the range of the literature values given for purple basil extracts.^67,68^ In addition, an LC-ESI-MS/MS study reported various anthocyanins with respective amounts, especially cyanidin derivatives (cyanidin-3-glucoside, cyanidin rutinoside), highly acylated anthocyanins that were found to be higher in the leaves of the purple basil plant compared to the flowers.^71,72^ Purple basil also contains a rich profile of bioactive compounds, including various flavonoids, such as quercetin diglucoside, quercetin rutinoside, and naringenin glucoside. It is also abundant in phenolic acids, rosmarinic acid as the dominant component, followed by chicoric, caftaric, and salvianolic acids.^71,72^ These compounds contribute to the plant’s strong antioxidant properties. As a consequence, purple basil is used frequently as a food additive, and it has great potential for use in food-related applications.

### 3.14 TAA Release of the Purple Basil Film

The release of the main components from the biopolymeric materials enriched with a natural extract is investigated to determine the loss in the material’s properties. The food freshness indicator property and potential food preservation ability of the prepared material depend on the anthocyanin content in the film. Thus, the release behavior of these compounds from the film into distilled water used as a water-containing food simulant was studied, and the release profile is shown in Figure 5. After 2 h, the release % reached the limit of 27.06%. It was understood that a significant amount of the anthocyanins was still in the film structure. Therefore, it can be assumed that the decrease in the contents of anthocyanins did not decrease the indicator properties of the film. The limited release of these compounds may also prove the interaction between the purple basil extract and the biopolymers via hydrogen bonding.^73^ Similar release trends were also reported for biopolymeric films prepared with different natural extracts.^73–75^

**Figure 5:**
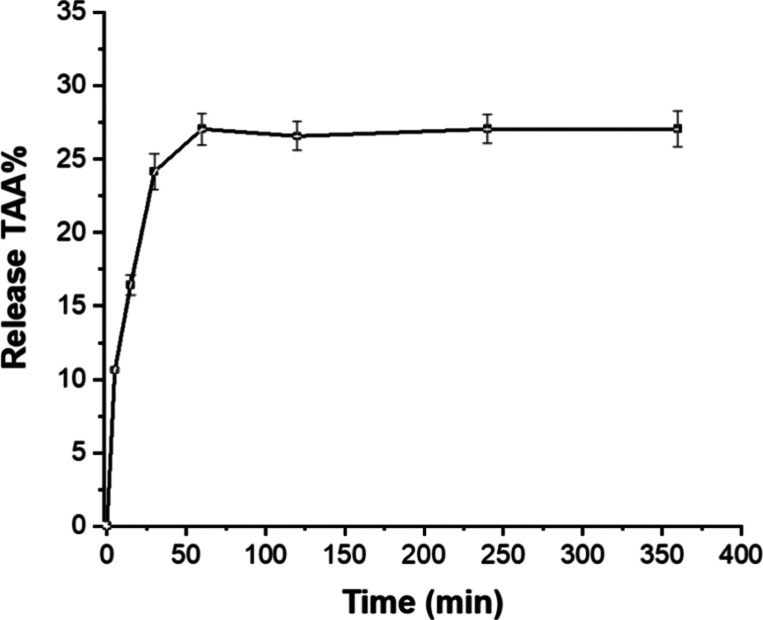
Total anthocyanin release % of the film prepared with purple basil extract.

The release profiles of the anthocyanins from the films were analyzed using different kinetic models, including first-order, Higuchi, Korsmeyer–Peppas, and Hixson–Crowell models, to better understand the release mechanism over 6 h and during the first hour of the initial release. Among the analyzed models, the Korsmeyer–Peppas model exhibited the highest coefficient of determination (*R* ^2^) value, indicating the best fit for the data, particularly for the initial release, with an *R* ^2^ value of 0.9741. The *R* ^2^ values and the regression equations of the models analyzed for both the 6 h period and the first hour are presented in Table 2. The model Korsmeyer–Peppas suggests that the release behavior is controlled by Fickian diffusion since the diffusion index (*n*) value is found to be ≤0.5.^76^

**Table 2:** Regression Equations and *R* ^2^ Values for Anthocyanin Release over 6 h and the First Hour

kinetic model	regression equation for 6 h	coefficient of determination (*R* ^2^) for 6 h	regression equation for 1 h	coefficient of determination (*R* ^2^) for 1 h
Hixson–Crowell	*y* = −0.0037*x* + 0.929	0.2152	*y* = 0.1325*x* + 2.3047	0.7772
first-order	*y* = 0.0016*x* + 2.890	0.3322	*y* = 0.0068*x* + 1.0776	0.7859
Higuchi	*y* = 1.1976*x* + 10.18	0.6175	*y* = 3.5933*x* + 1.7629	0.9616
Korsmeyer–Peppas	*y* = 0.2071*x* – 1.051	0.7712	*y* = 0.394*x* + 0.7591	0.9741

### 3.15 Antibacterial Activity of the Prepared Film

After 6 h of contact time, enumeration results showed that the film samples containing purple basil extract had a significant antimicrobial effect on both bacterial species, with a reduction of more than 1 log. In Figure 6, the results are compared with the control plain film of PP, control film (film prepared without extract), and film prepared with purple basil extract. Previous studies have shown that purple basil extracts and essential oils have a broad-spectrum antibacterial effect.^77,78^ The film prepared without extract has limited antibacterial ability compared to the film obtained with the extract as well as antioxidant activity and phenolic content. Thus, the developed film has a high potential for food-related applications.

**Figure 6:**
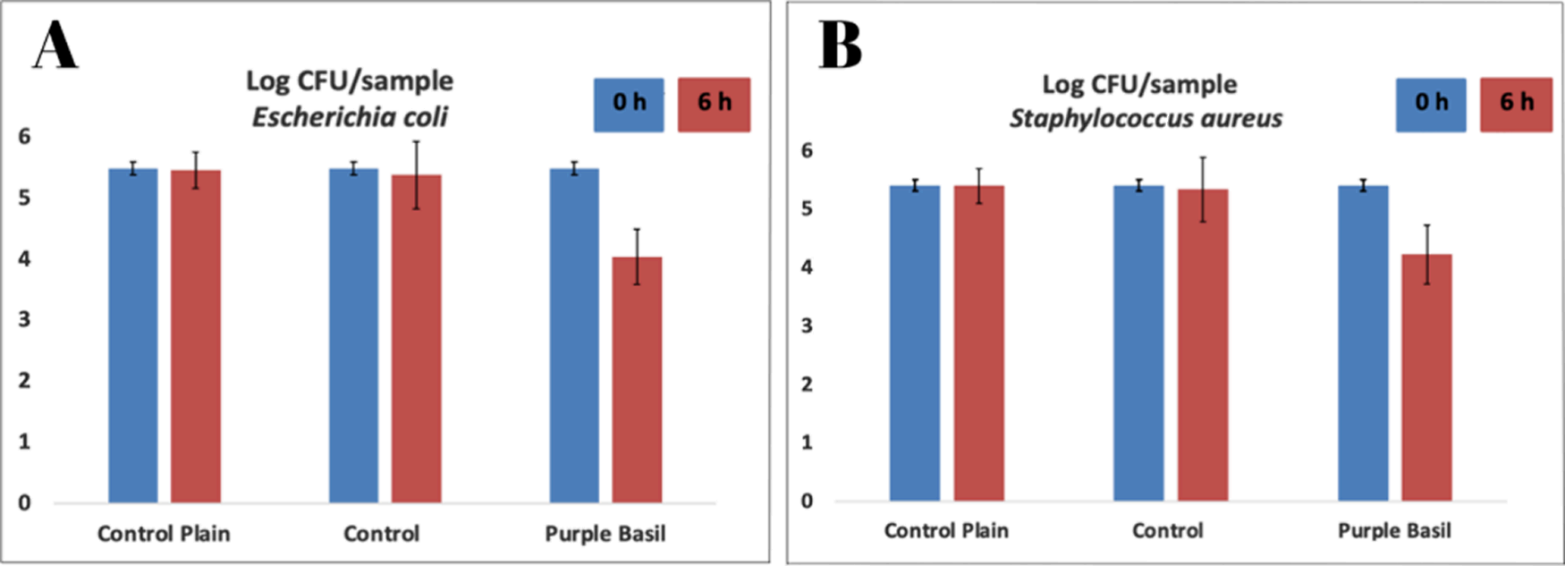
Antimicrobial activities of the film prepared with and without purple basil extract against bacterial species (A) Escherichia coli and (B) Staphylococcus aureus.

### 3.16 Color Changes of the Purple Basil Extract with pH

The color transitions of anthocyanin-containing extracts depend on the amount and type of anthocyanins they contain, but in general, the red color of the flavylium cation at acidic pH levels passes through violet color at neutral pH levels and turns into a blue anionic species around pH 8. After pH 11, the color becomes green and gradually yellow green.^79^ Although there may be differences in color tones, the color change in the acidic and basic regions is noticeable enough to be seen with the naked eye. Changes in the anthocyanin structure and colors at different pH levels are given in Figure 7A.

**Figure 7:**
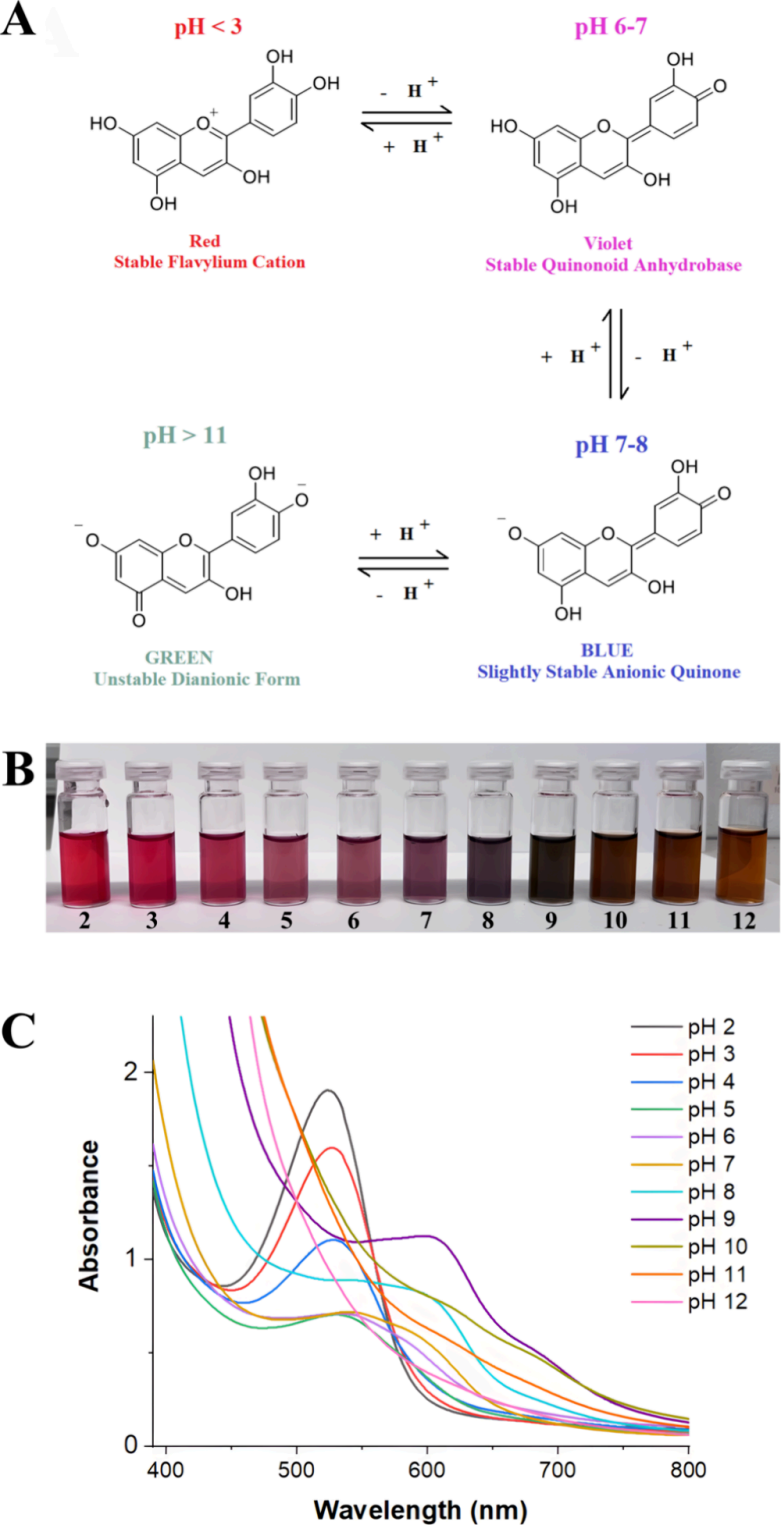
(A) Structural and color changes of the anthocyanins at different pH values. (B) Visual color changes of the purple basil extract between pH 2.0 and 12.0. (C) UV–Visible spectra in the 400–800 nm wavelength range of the purple basil extract in different pH values ranging from 2.0 to 12.0.

The color sensitivity of basil extract regarding the pH changes between 2.0 and 12.0 was recorded and is described in Figure 7B. The UV–Visible spectra of the extract in different pH levels are given in Figure 7C. At lower pH values, the maximum wavelength was around 520 nm. In contrast, with an increase in the pH, the maximum wavelengths were shifted to higher values around 595 nm, and the peak intensities were decreased. The recorded changes proved the sensitivity of the anthocyanin-containing extract to changing pH and its potential for use as a pH indicator as resembling other studies.^9,64,65,80^

### 3.17 Color Changes of the Composite Biopolymer Film with pH

The films containing purple basil extract were cut into rectangular pieces and immersed in buffer solutions with different pH. The color changes were then meticulously recorded and are presented in Figure 8. The distinct red-pink color of the film, a hallmark of the acidic environment, transformed into a vibrant violet at pH 6. A striking blue color at pH 8 followed this. Beyond this point, a deep green shade dominated the films.

**Figure 8:**
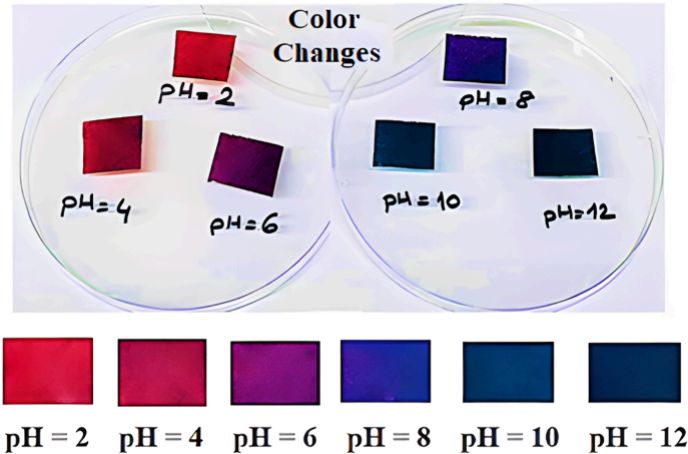
Digital images of the color changes of the pH-sensitive film in various buffer solutions.

Food spoilage is the term used for foods that are no longer safe to consume due to some chemical, microbial, or physical changes.^81^ Protein-rich foods undergo spoilage depending on several factors, such as pH, the contents and structure of food, and storage conditions.^82^ During deterioration, microbial growth and proteolysis occur. Microorganisms use the peptides and free amino acids formed by proteolysis, and then the products, such as volatile amines, form.^83^ When volatile alkaline compounds spread into the smart film due to the decay of high-protein foods, an alkaline environment is created, causing the film’s anthocyanin to change color. Figure 8 shows that when the films are basic with volatile amines formed due to food spoilage, the color transformation is visible to the naked eye.

### 3.18 Application of the Film to a Real Food Sample as a Spoilage Indicator

Foods that are no longer safe to consume due to some chemical, microbial, or physical changes are considered spoiled.^81^ Both manufacturers and consumers share concerns about food safety and promote the necessity of intelligent packaging materials to prevent the consumption of spoiled food. The applicability of the prepared material as an indicator of food spoilage was tested with raw poultry. Chicken breast, which stated on the label that it could be stored for a week under refrigerator conditions, was purchased on the first day of arrival at the market. The piece of film was placed on the inner surface of the chicken breast package, and the chicken breast was kept in the refrigerator at 4 °C. The bluish-violet color transformation of the film became visible on the seventh day before its expiration date. The film turned green after 15 days, and the meat’s deterioration was also evident. The visual changes in both the color of the film and the meat are represented in Figure 9. The changes in color properties of the films are represented in Table 3 with the color properties of the original film and the film at the end of the complete color change. The redness (*a*) value was significantly decreased, as expected, due to the color change from purple to green. Δ*E*, the color difference from days 0 and 15, was calculated and represented, indicating the pH-sensitive color-changing ability of the prepared material.

**Table 3:** Color Properties of the Original Film Compared with the Film Used for Indicator Purposes^a^

sample	L* ± SD	a* ± SD	b* ± SD	C* ± SD	WI ± SD	Δ*E* ± SD
original film	28.37 ± 0.32	10.07 ± 0.40	–2.18 ± 0.66	10.32 ± 0.51	29.67 ± 7.00	
the film piece was stored with meat at 4 °C	28.71 ± 0.80	2.27 ± 0.04	–1.19 ± 0.86	2.63 ± 0.40	19.14 ± 9.60	63.21 ± 6.82

a ±Standard deviations (SD), *n* = 3, L*: lightness, a*: redness, b*: yellowness, C*: chroma, WI: whiteness index, Δ*E*: color difference.

**Figure 9:**
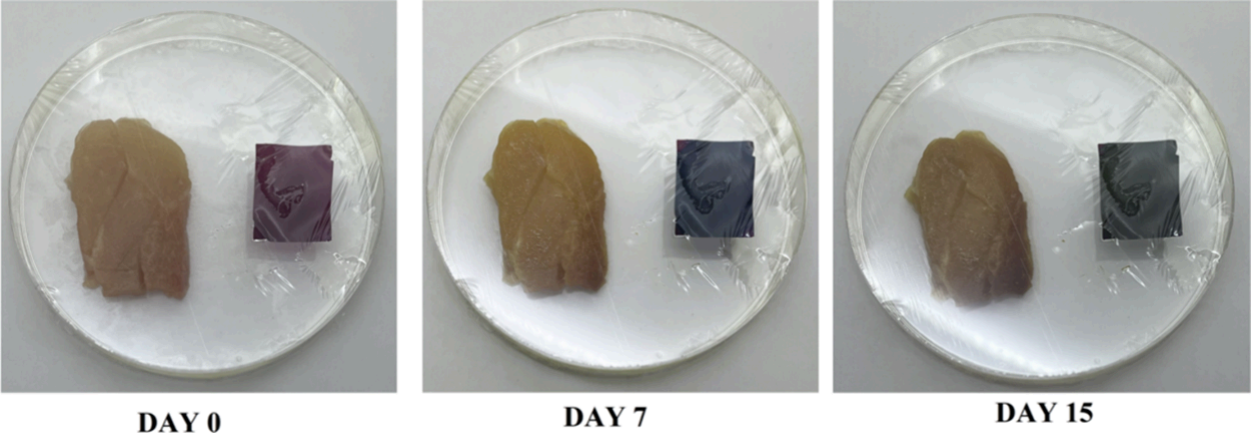
Color change of the film prepared with the purple basil extract stored in the refrigerator at 4 °C.

## Conclusions

In this study, a novel, active, and intelligent composite material from alginate and pectin biopolymers incorporated with a purple basil extract was prepared. The physical, antioxidant, and antimicrobial properties of the films were examined, specifically focusing on their potential application as food packaging materials. The results indicated that the film demonstrated significant antioxidant and antibacterial activities, effectively inhibiting both Gram-positive and Gram-negative bacterial species. This antimicrobial effect suggests the potential of the film to extend the shelf life of food products by controlling the bacterial growth. Furthermore, the films’ anthocyanin content showed a successful color-changing nature, making it possible to detect food spoilage. This effectiveness was proven through application to real chicken breast meat. The observed color change of the film provides an easy and reliable indication of spoilage and highlights the potential usage as a responsive food packaging material, which also prolongs the product’s shelf life due to its antioxidant and antibacterial activity. The physical properties of the materials were also investigated. The thermal stability, which is essential during material processing, was observed alongside the impressive UV-light-blocking ability of the film. The UV-light-blocking ability is essential for protecting sensitive food products from oxidative degradation, increasing the material’s potential to be used for a wide range of foods that are sensitive to UV-light exposure. The simple, straightforward, and cost-effective preparation of the material, combined with its environmental benefits, shows a promising, active, and intelligent biopolymer-based packaging system. This material not only preserves food but also promotes sustainability and consumer awareness regarding food quality.
